# Bilateral Pulmonary Arteriovenous Malformations: Challenges in a Rare and Complex Case

**DOI:** 10.7759/cureus.70231

**Published:** 2024-09-26

**Authors:** Aayushi Joshi, Vasu Saini, Bhumika Bheemavarapu, Anjani Mahesh Kumar Cherukuri, Mohd. Shaban

**Affiliations:** 1 Pediatrics, Shri Guru Ram Rai Institute of Medical and Health Sciences, Dehradun, IND; 2 Pediatrics, Jawaharlal Institute of Postgraduate Medical Education and Research, Pondicherry, IND; 3 Pediatrics, Guntur Medical College, Guntur, IND

**Keywords:** congenital abnormalities, congenital birth defect, congenital disease, endovascular coil embolization, hereditary hemorrhagic telangiectasia (hht), pavm, perioral cyanosis, pulmonary arteriovenous malformations

## Abstract

Pulmonary arteriovenous malformations (PAVMs) are abnormal vascular connections between the pulmonary arteries and pulmonary veins. Despite their relatively uncommon incidence, PAVMs should be considered in the differential diagnosis of children presenting with cyanosis due to the life-threatening complications posed by paradoxical emboli. The primary management approach involves eliminating the abnormal connections, either through surgical or endovascular methods. We present a case of a five-year-old boy who was seen in the Outpatient Department with cyanosis of the lips and nail beds persisting for eight months. On examination, grade 3 clubbing was noted. Imaging and subsequent angiography revealed multiple bilateral diffuse AVMs, which were treated using an endovascular approach.

## Introduction

Pulmonary arteriovenous malformations (PAVMs) involve abnormal connections of blood vessels that create a right-to-left (R-L) shunt between the pulmonary artery and vein, allowing blood to circumvent the pulmonary capillaries. This anomaly can disrupt gas exchange, leading to lower oxygen levels and shortness of breath, especially during exertion. Moreover, the abnormal and frequently fragile walls of these vessels may make individuals more susceptible to pulmonary bleeding [1]. This anatomical right-to-left shunt permits emboli and bacteria to bypass the pulmonary capillary bed, potentially leading to severe complications, including pulmonary artery hypertension, high-output cardiac failure, stroke, transient ischemic attack, or cerebral abscesses [2].

PAVMs are more prevalent than commonly recognized, affecting approximately 1 in 2,500 individuals (95% CI, ranging from 1 in 1,300 to 1 in 5,600) [3]. Notably, they are more frequently observed in females [4]. Furthermore, over 80% of these cases are associated with hereditary hemorrhagic telangiectasia (HHT) [5]. Accurate diagnosis and effective management are imperative, given that PAVMs can result in substantial morbidity and mortality. The diagnosis of PAVMs is usually confirmed by relying on clinical and radiological assessments. Presently, multidetector CT is deemed the most accurate and least invasive diagnostic technique available [6]. The mainstay of treatment for PAVMs is the removal of the abnormal blood vessels, accomplished through either open surgery or endovascular techniques.

## Case presentation

**Figure 1:**
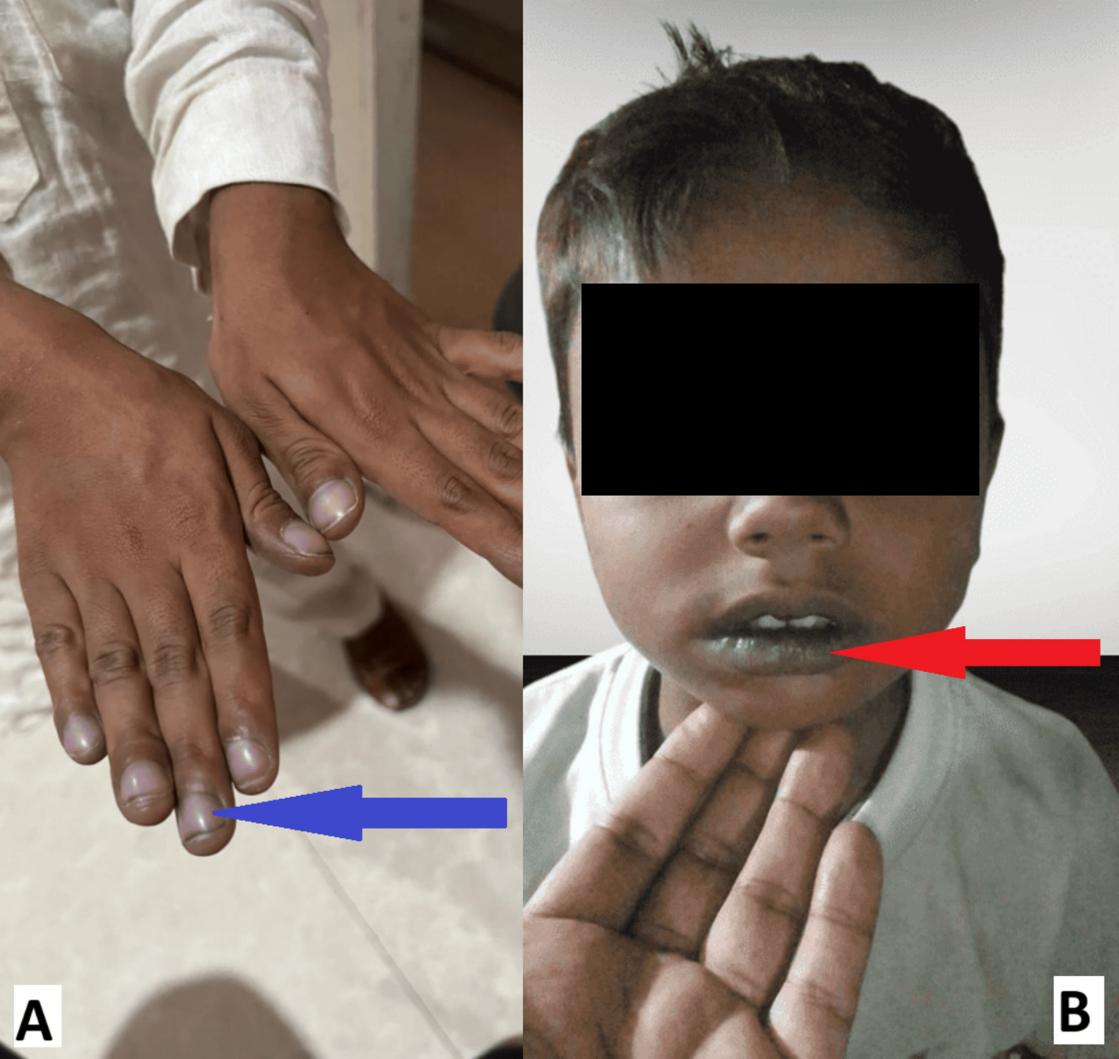
(A) Clubbing with cyanosis of the nail beds (blue arrow) and (B) cyanosis of the lips (red arrow)

A five-year-old boy, with a normal birth and developmental history, of Muslim faith, born to non-consanguineous parents and hailing from the northern part of India, presented to the pediatric outpatient department with complaints of intermittent episodes of cyanosis involving the oral mucosa and nail beds (Figure 1) along with intermittent episodes of dyspnea for eight months, which were aggravated on exertion (NYHA class III), and associated with excessive sweating upon exertion. On examination, his heart rate was 92 beats per minute, respiratory rate was 24 breaths per minute, and blood pressure was 96/40 mm Hg. He was afebrile, with oxygen saturation measurements of the right upper limb at 74%, right lower limb at 71%, left upper limb at 78%, and left lower limb at 68%. General examination revealed cyanosis of the lips and nail beds with grade 3 clubbing (Figure 1). Cardiac examination was unremarkable with normal S1, S2, and no murmurs on auscultation, and the rest of the systemic examinations were normal.

At the time of admission, laboratory results showed polycythemia (hemoglobin level of 20 g/dl, a red cell count of 9.6 million cells per cubic millimeter, and an increased hematocrit value of 71.5%). A peripheral smear revealed significant anisopoikilocytosis, with predominantly normocytic normochromic cells, a few microcytic hypochromic cells, and a reticulocyte index of 2.8. Leukocyte and platelet counts were within normal ranges. Kidney function tests, liver function tests, and serum electrolytes were normal (Table 1).

**Table 1:** Investigation results BUN: blood urea nitrogen; SGOT: serum glutamic-oxaloacetic transaminase; SGPT: serum glutamic-pyruvic transaminase; P: polymorphonuclear cells; L: lymphocytes

Parameters	Results	Normal range
Hemoglobin (gm/dl)	20	11.5-13.5
Red cell count (million cells per cubic millimeter)	9.6	4.0-5.5
Hematocrit (Hct)	71.5%	34-40%
Total count(/mcl)	5700	4000-11000
Differential count	P48%, L31%	P40-60%, L20-40%
Platelet count (/mcl)	215000	150000-450000
Serum bilirubin (total mg/dl)	0.8	0.1-1.2
SGOT (iu/l)	28	5-40
SGPT (iu/l)	45	7-56
BUN (mg/dl)	18	7-20
Serum creatinine (mg/dl)	0.6	0.4-0.7

**Figure 2:**
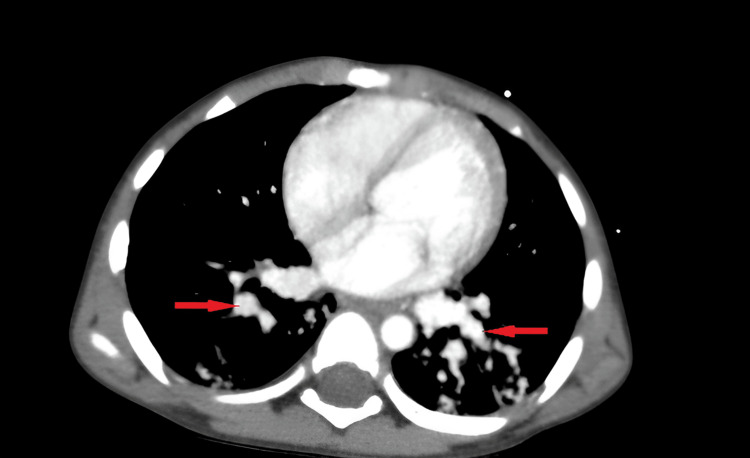
Homogeneously enhancing, well-circumscribed, lobulated vascular lesions (red arrow) in the bilateral lung fields

**Figure 3:**
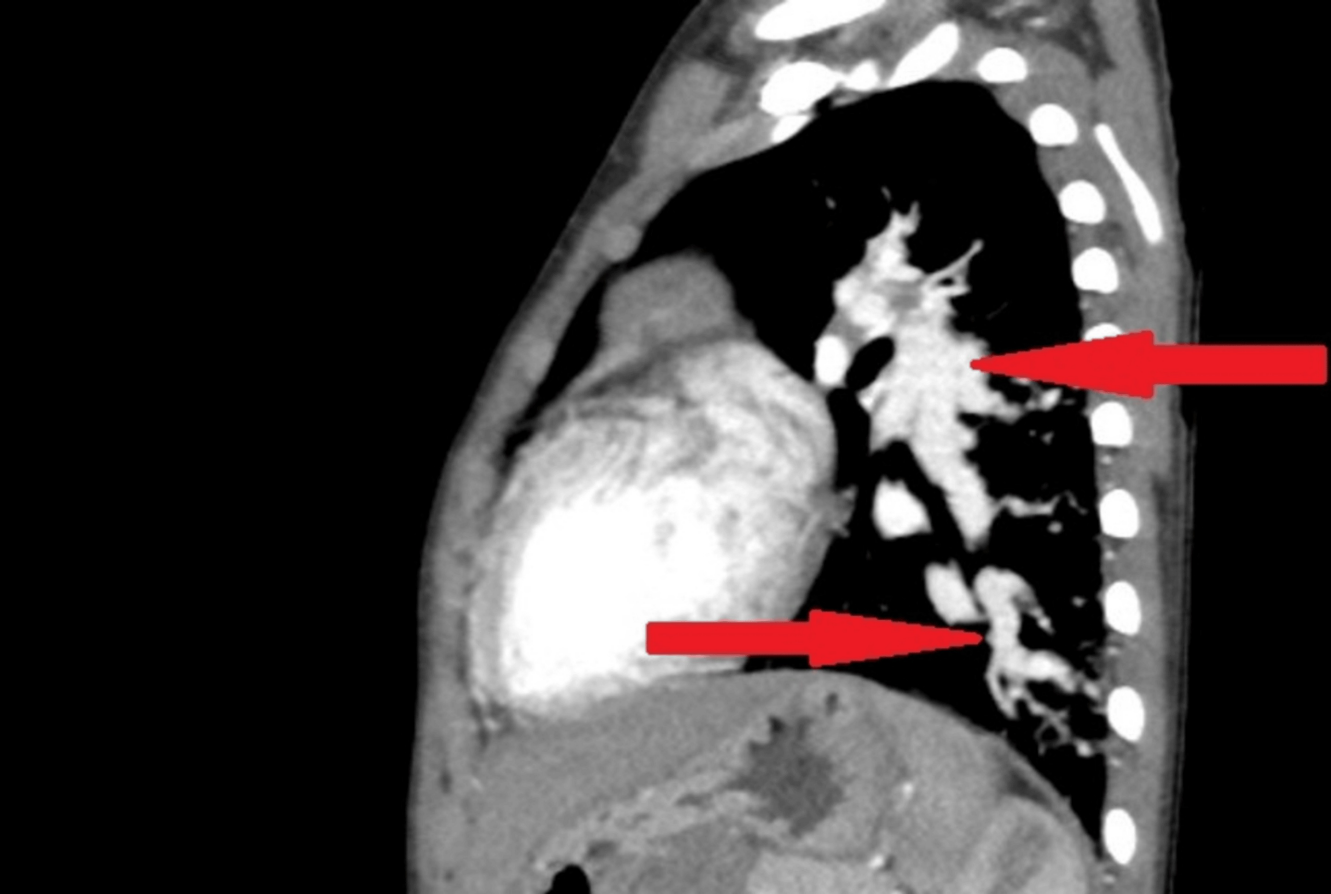
CT angiography of the thorax revealing multiple pulmonary arteriovenous malformations (red arrow)

The two-dimensional echocardiogram was unremarkable. CT angiography of the thorax revealed multiple PAVMs along with aorto-pulmonary collaterals in the bilateral lung parenchyma (Figures 2, 3).

The child was treated symptomatically with oxygen inhalation via a nasal cannula at a rate of 4 liters per minute and underwent a partial volume exchange transfusion to address elevated hematocrit (Hct) levels. Using the formula: Blood volume x body weight x (observed Hct - desired Hct)/observed Hct, 185 mL of blood was exchanged for an equivalent volume of normal saline. Pulmonary angiography was done and multiple small AVMs in the bilateral lungs were found. Small AVMs in the left lingula were identified, and coiling with embolization was attempted. However, saturation did not improve significantly, although the difficulty in breathing improved considerably. In view of multiple bilateral AVMs arranged in a diffuse manner, further procedures were abandoned.

## Discussion

PAVMs are primarily congenital anomalies of the terminal capillary loops. They can also be associated with various systemic conditions, including HHT, hepatic cirrhosis, schistosomiasis, mitral stenosis, and Fanconi syndrome, as well as trauma or post-cardiac surgical procedures [7]. The exact cause of pulmonary AVMs is not fully understood. Recent genetic studies on HHT have identified germline mutations in two key genes, ENG and ACVRL1, involved in the transforming growth factor-beta pathway [1,8]. These mutations may be linked to the development of abnormal connections between the pulmonary arteries and veins.

PAVMs are classified into simple and complex types based on the number of feeding pulmonary arteries. The simple type is the most common, making up about 80% of cases, and consists of a lesion with a single feeding pulmonary artery and one draining pulmonary vein. In contrast, a complex PAVM is characterized by multiple feeding arteries. A rare subtype of the complex type, known as diffuse PAVM, can affect all segments of the lung or, less frequently, an entire lobe [5]. It is important to recognize that various vascular pathologies affecting the lung, pleura, and mediastinum can present similarly to PAVMs both clinically and in imaging studies. These conditions include extrapulmonary AVMs, anomalous pulmonary veins, pulmonary vein atresia or stenosis, partial anomalous pulmonary venous drainage, pulmonary vein varix, and pulmonary artery aneurysms [9]. While a detailed analysis of contrast-enhanced CT can often yield a precise diagnosis, pulmonary vessel angiography remains definitive when imaging alone does not provide sufficient clarity. The fundamental strategy for managing PAVMs is to eliminate the abnormal blood vessels through either an open surgical technique or an endovascular approach, the latter of which generally involves the use of vascular plugs, silicon balloons, or metal coils [2,10]. 

Minimally invasive endovascular embolotherapies are considered the primary treatment of choice, as they can avoid a myriad of peri-operative complications associated with the surgical approach [2]. It is important to note that embolotherapies have their own complications, such as air embolism, pulmonary infarction, pleuritic chest pain, recanalization, and reperfusion of the AVM, migration of the embolic device, and the interval growth of new PAVMs [1,4,6]. It was observed that employing vascular plugs for embolization is associated with significantly lower recanalization rates in comparison to coils [11]. Although it was previously the norm to employ a surgical approach for large and diffuse AVMs, recent studies suggest that embolization should be attempted even in large AVMs owing to the complications associated with the surgical approach [12]. Timely intervention, in addition to regular follow-up, can improve the morbidity and mortality associated with PAVMs.

## Conclusions

Although rare, PAVMs should be considered in the differential diagnosis of cyanosis in a child, as they are associated with severe complications arising from paradoxical emboli. Individuals with HHT should be screened regularly as they are at a higher risk of developing PAVMs. Timely intervention can help prevent the progression of PAVMs and avoid complications such as pulmonary artery hypertension, high-output heart failure, stroke, or cerebral abscesses associated with them.
